# Results of Treatment of Squamous Cell Carcinoma of Maxillary Sinus: A 26-Year Experience

**DOI:** 10.4021/wjon2010.02.191w

**Published:** 2010-02-01

**Authors:** M. Ashraf, J. Biswas, A. Dam, A. Bhowmick, Jha, V. Sing, S. Nayak

**Affiliations:** a Department of Surgical Oncology, Chittaranjan National Cancer Institute (CNCI), 37, S P Mukherjee Road, Kolkata-26, India; b Department of Head and Neck Surgery, Chittaranjan National Cancer Institute (CNCI), 37, S P Mukherjee Road, Kolkata-26, India

**Keywords:** Squamous, Antrum, Maxillary, Second primary

## Abstract

**Background:**

Five-year survival in squamous cell carcinoma of maxillary antrum is low. This article examines the results of various approaches to treatment as given in our hospital in past 26 years.

**Methods:**

From 1979 to 2005, 379 patients with squamous cell carcinoma of maxillary antrum managed with curative intent were studied. Twenty-eight patients had T2, 237 patients had T3, and 114 had T4 tumors. The N classification was N0 in 316 patients, N1 in 21 patients, N2a in 28 patients and N2b in 14 patients. Treatment to the primary site comprised of surgery (Sx) and radiation therapy (RT) in 284 patients, RT alone in 57 patients and chemotherapy (CTx) with radiotherapy in 38 patients.

**Results:**

There was a difference in survival between patients who underwent Sx with RT compared with patients who received RT alone or CTx with RT. The most common pattern of recurrence was in the primary site, 187 (49.3%) patients. Local control at 3 and 5 years was 71% and 63.8% respectively in Sx with RT, 31.6% and 28% respectively in RT, and 28.9% and 26% in CTx with RT group.

**Conclusions:**

The type of treatment to the primary site is an important determinant of survival and local control. Surgery with radiation is a better treatment option.

## Introduction

Maxillary sinus cancer is relatively rare neoplasm, with an incidence representing a small percentage (0.2%) of human malignant tumours and only 1.5% of all head and neck malignant neoplasms [1, 2]. Because of the relative rarity of carcinoma of the maxillary sinus, institutional experience is usually limited. The incidence seems to vary in different parts of the world, with Asian countries reporting high numbers of cases [3-5]. However, the experience with regard to the incidence and presentation in Western Europe and the United States is similar. Carcinomas of the maxillary sinus comprise nearly 80% of all cases of paranasal sinus tumors [6-9]. In a fairly good majority of patients, this cancer is diagnosed in advanced stages, making it difficult to determine the origin of the neoplasm [9]. Although the majority of maxillary sinus carcinomas are locally advanced at diagnosis because its symptoms are nonspecific and resemble sinusitis [2], yet these tumours tend to remain localized to maxilla for a long time and during evolution they invade adjacent structures such as bone, base of the skull, facial soft tissue, oral cavity, and orbits[2, 6, 7, 10-13]. Treatment of these tumors has included either surgery, radiation therapy, or a combination of both. Nevertheless, in more advanced stages, both surgery and radiotherapy have limitations [2, 6, 9, 14]. The purpose of this article was to review our experience with maxillary sinus carcinoma and to examine the results of various approaches to treatment as given in our hospital.

## Materials and Methods

### Patients and tumor characteristics

From 1979 to 2005, 379 patients with squamous cell carcinoma of maxillary antrum were managed with curative intent in the departments of surgical oncology, ENT and radiation oncology of Chittaranjan National Cancer Institute, Kolkata, India. There were 274 (72.2%) male and 105 (27.8%) female patients with a mean age of 47.2 years (range 23 - 67 years). Tumor was on the right side in 236 (62.2%) patients and on the left side in 143 (37.8%) patients. The location of the neoplasm was suprastructure only in 69 (18.2%) patients, infrastructure only in 142 (37.5%) patients, and both in 168 (44.3%) patients .The tumors were well differentiated in 132 (34.9%) patients, moderately differentiated in 182 (48%) patients, and poorly differentiated in 65 (17.1%) patients. All tumors were radiologically assessed. Between 1979 and 1987, tomograms were used. Since 1988, computerized tomography (CT) had been introduced, and since 1991, magnetic resonance imaging (MRI) has been utilized, specifically in cases where additional information with regard to soft tissue extension, such as in the orbit or retro-maxillary and intracranial regions, was required. Tumor extension was found on an average of 1.4 adjacent sites (range 1 to 4 adjacent sites). Frequency of invasion was highest into the tissue underlying the skin (134 patients, 35%) followed by involvement of ethmoid sinuses (98 patients, 25.8%) and skin of the cheek (95 patients, 25%). Tumor extended to the orbit in 37 patients (9.8%), eye in 34 patients (9%), cribriform plate in 52 patients (13.7%), pterygoid fossa in 55 patients (14.5%), sphenoid sinus in 26 patients (6.8%), and infra temporal fossa in 26 patients (6.8%). Tumors were classified retrospectively using the American Joint Committee on Cancer Staging System [15]; tumor classification was T1 in 0 patients, T2 in 28 patients, T3 in 237 patients, and T4a in 114 patients (Table 1). Lymph nodes were positive in 63 patients (16.6%). N classification was N0 in 316 patients, N1 in 21 patients, N2a in 28 patients, N2b in 14 patients, and no patient had N2c nodes.

**Table 1:** Tumor and Lymph Node Stage in 379 Patients With Maxillary Sinus Carcinoma According to Treatment of the Primary Site

Stage	Sx and RT* (no. of patients)	RT and Sx* (no. of patients)	RT (no. of patients)	CTx* and RT (no. of patients)	Total
T2N0	18	0	10	0	28
T3N0	151	25	38	2	216
T3N1	4	13	0	4	21
T4N0	16	14	19	23	72
T4N1	0	0	0	0	0
T4N2a	12	7	0	9	28
T4N2b	14	0	0	0	14
Total	225 (59.4%)	59 (15.6%)	57 (15%)	38 (10%)	379 (100%)

*Abbreviations: Sx - Surgery; RT - Radiation Therapy; CTx - Chemotherapy.

Treatment to the primary site comprised surgery and radiation therapy in 284 patients, radiation therapy alone in 57 patients and a combination of chemotherapy and radiation in 38 patients. Indications for nonsurgical therapies were patients’ age, medical status including surgical risk, and the patients’ choice, the later being the predominant factor which decided the nature of treatment. Chemotherapy consisted of methotrexate and/or bleomycin and/or cisplatin, three to four courses, followed by megavoltage radiotherapy in daily doses of 2 Gray, 5 days a week, to a total of 60 to 65 Gray in 30 to 32 sittings. Radiation was delivered through two wedge pair or three fields (one anterior and two laterals) with customized blocks to prevent irradiation of normal tissues, with special attention to the orbital contents and the optic nerve. The median dose to the primary site for patients treated with radiotherapy alone was 7000 cGy using fractions of 180 - 200 cGy. The regional lymph nodes were treated only in patients with neck involvement. The tumor and neck stage for patients undergoing surgery and radiation therapy, chemotherapy with radiotherapy and radiotherapy alone is presented in Table 1. In the patients who underwent surgery (n = 284), 225 received postoperative radiotherapy and 59 received preoperative radiotherapy. Surgical resection was described as a total maxillectomy in 196 patients, partial maxillectomy in 63 patients, completion maxillectomy in 25 patients and local excision of recurrent nodules in 39 patients. 34 patients underwent an orbital exenteration and 63 underwent a neck dissection. For patients receiving postoperative irradiation, radiotherapy usually was started 3 - 6 weeks after surgery. The dose to the primary site for patients receiving postoperative radiotherapy was 5500 centigray (cGy) for tumors with margins more than 1 cm free of tumor, 6000 cGy for tumors with less than 1 cm margin free of disease, and 6600 cGy for tumors with positive margins. For patients receiving preoperative radiotherapy the dose was 5000 cGy.

### Statistical analysis and follow-up

For calculation of survival and local control, endpoints were determined from the date of diagnosis until the event of interest. For survival, the event of interest was death. For disease free survival, the event of interest was local, regional, or distant failure. For local control, the event of interest was local failure. The mean and median follow-up times were 93 and 67 months, respectively (range 37 - 365 months).

## Results

### Survival

The 3-, 5-, and 10-year overall survival rates of patients with maxillary sinus carcinoma were 61.5%, 53% and 36.9% respectively. For patients who underwent surgery and postoperative radiation therapy, the 3-, 5- and 10-year overall survival rates were 69%, 63.5% and 48% respectively. For patients who received preoperative radiotherapy and surgery, the 3- and 5-year survival rates were 54.3% and 47% respectively, and the 10-year survival rate was 27%. For patients who received radiation therapy alone, the 3- and 5-year survival rates were 47% and 31% respectively, and 10-year survival rate was 19%. For patients who received chemotherapy and radiation, the 3- and 5-year survival were 47.4% and 34% respectively, and the 10-year survival was 13%. Comparison of overall survival according to treatment to the primary site is shown in Table 2.

**Table 2:** Survival and Local Control Data According to Treatment of the Primary Site

Treatment	3-year		5-year		10-year Survival (%)
	Survival (%)	Local control (%)	Survival (%)	Local control (%)	
Sx and RT* (n = 225)	69%	71%	63.5%	65.8%	48%
RT and Sx* (n = 59)	54.3%	50%	47%	38.9%	27%
CTx* and RT (n = 38)	47.4%	28.9%	34%	26.3%	13%
RT (n = 57)	47%	31.6%	31%	28%	19%

*Abbreviations: Sx - Surgery; RT - Radiation Therapy; CTx - Chemotherapy.

### Patterns of recurrence

The patterns of recurrence for all 379 patients were as follows: the most common site of recurrence was in the primary site, which was observed in 187 of 379 patients (49%); failure in the regional lymph nodes and distant sites were observed in 96 (25%) and 43 (11.4%) patients respectively; isolated local failure was the most common pattern of recurrence, as demonstrated in 107 of 379 patients (28.2%).

### Local control

The 3-, 5-, and 10-year actuarial estimates of local control for all patients were 57.8%, 52% and 44.2% respectively. For patients who underwent surgery and postoperative radiation therapy, the 3- and 5-year local control rates were 71% and 65.8% respectively; for patients who received preoperative radiation therapy and surgery, the 3- and 5-year local control rates were 50% and 38.9% respectively; for patients who received chemotherapy and radiation, the 3- and 5-year local control rates were 28.9% and 26.3% respectively; and for patients who received radiation therapy alone, the 3- and 5-year local control rates were 31.6% and 28% respectively. There was a statistically significant (p = 0.0002) difference in local control rates between surgery with postoperative radiotherapy and other (radiotherapy alone, chemotherapy with radiation, and preoperative radiation followed by surgery) groups.

### Complications

There were a total of 196 documented complications. The most common type of complication was the formation of fistulae, which were found in 73 (19.3%) patients, of which 38 were from the group of patients who had received preoperative radiation therapy (n = 59, 64%) and 35 patients from the group who had received postoperative radiation and developed oro-cutaneous fistula (n = 225, 15.6%). Thirty-seven patients (9.7%) developed cataracts, 10 patients (2.6%) developed retinopathy, 23 patients (6%) developed epiphora, 12 patients (3.2%) developed nasal obstruction, and 5 patients (1.3%) developed cellulitis. Osteoradionecrosis of the orbital wall was found on average 6.6 years (range 3 to 9 years) after radiotherapy in 8 patients (2%).

### Second cancers

Second primary tumors were documented in 19 (5%) patients. Four patients had poorly differentiated, 8 patients had well differentiated and 7 patients had moderately well differentiated squamous cell cancers on histopathology. In 9 out of these 19 patients, the tumors arose in the gingivo-buccal sulcus, in 5 patients from dorsal tongue, and in 3 patients from larynx. In one patient each tumor arose from floor of the mouth and tonsil. Average interval from the time of diagnosis of primary tumor and the appearance of second primary malignancy was 7.68 years (range 4.5 to 11.5 years). All these patients had received radiation ranging from 5500 cGy to 7000 cGy. T staging of these tumors was T2 in 14 patients and T1 in 5 patients. All were managed by surgery .There was no mortality associated with second primary tumors.

## Discussion

Maxillary sinus carcinoma presents a therapeutic challenge to both the surgeon and the radiation oncologist. Because symptoms are vague and nonspecific, the majority of carcinomas are diagnosed as locally advanced disease [2]. Extension to contiguous structures including the orbit, ethmoid sinus, sphenoid sinus, nasal cavity, nasopharynx, pterygoid fossa, palate and cheek may occur and can be a potential problem in the surgical and/or radiotherapeutic management of this disease [2, 9]. In our study, frequency of invasion was highest into the tissue underlying the skin (134 patients, 35%) followed by involvement of ethmoid sinuses (98 patients, 25.8%) and skin of the cheek (95 patients, 25%). Tumors were predominantly T3 or T4 as observed in 351 of 379 patients (92.6%). Similar incidence has been presented from other parts of the world [4, 5, 8, 9]. Sakai et al [3] reported a series of 773 patients with maxillary sinus carcinoma seen in a 22-year period between 1959 and 1979. Rifki [4] reported that carcinoma of the maxillary sinus in Indonesia is the second most common malignancy in the head and neck, only next to nasopharyngeal carcinoma. From India, Sharma et al [5] reported 226 cases of carcinoma of the maxillary sinus in a 10-year period. The same authors reported a 40.7 % incidence of cervical nodal metastasis at first presentation. In our study lymph nodes were positive in 63 patients (16.6%). Most series report an incidence of about 10% (range 7% to 22%) of nodal metastasis in the cancer of maxillary antrum [11, 16, 17]. The striking difference in incidence and mode of presentation of patients, guess Tiwari et al [18], in western countries and some Asian and African countries suggests that we are dealing with a histologically similar tumor with different biological behavior. It may be that the etiological factors between the two groups are also different.

Local recurrence is an important cause of failure of the curative treatment of maxillary sinus carcinoma [2, 18, 19]. Pattern of failure analysis in 379 consecutive patients seen at our institution for curative treatment revealed that 49% of patients failed at the primary site. Isolated local failure occurred in 28.2% of patients and was the most common pattern of recurrence. Therefore, strategies to improve local control are imperative in the management of this disease. Analysis of the treatment characteristics in the local control of maxillary sinus carcinoma revealed that the type of treatment to the primary site was a significant variable. Patients who underwent a combination of surgery and adjuvant radiation therapy had a 5-year local control rate of 63.8% compared with 38.9%, 28%, and only 26% respectively, for those who received neoadjuvant radiation therapy and surgery, radiation alone, and a combination of chemotherapy and radiation. Table 2 shows the local control rates in different series in patients who underwent surgery and radiation therapy versus patients who received radiation therapy alone and chemotherapy with radiation. In general, our results are comparable to the reported results of other authors in that, the treatment by surgery and radiation therapy yields local control rates of 53 - 78% whereas local control after radiation therapy alone ranges from 14% to 55% [2, 6, 9-11, 16, 20-22].

Considering the retrospective nature of our study and a relatively small number of patients (n = 59) who received preoperative radiation, the question of whether radiation therapy should be given before or after surgical resection cannot be addressed definitively in our study. Radiation therapy has been proposed to be given preoperatively because the blood supply to the tumor remains intact and hence tumor hypoxia is less of a problem, at least theoretically. Yu Hua et al [23] reported a 64% 5-year survival rate in the preoperative radiation therapy group and a 5-year survival rate of 26% in the postoperative radiation therapy group. The report suggests that the complication rate was higher in the preoperative group compared with patients who received postoperative radiation therapy (29% versus 14%). Older studies like that of Jesse, however, report no difference in local control or survival. In a comparative study by Jesse, of preoperative and postoperative radiation therapy in patients with squamous cell carcinoma of the paranasal sinuses, patients who were treated with preoperative radiation therapy had a higher morbidity rate [24]. A more recent report by Guo et al, from China, supports the use of postoperative irradiation and shows a 5-year survival rate of 50.8% in 151 patients with maxillary sinus carcinoma [25]. Our results indicate a lower 5-year local control rate (38.9%, n = 59) and higher postoperative complication rate (64% fistula rate) in preoperative radiation group of patients as compared to 5-year local control rate of 63.8% (n = 225) and postoperative complication rate of 15.6% (oro-cutaneous fistulae) in postoperative radiation group.

Chemotherapy in the form of Cisplatin was introduced only in the 1980s [26]. Until that time, Methotrexate was the drug used in squamous cell carcinoma. Extensive use of combination chemotherapy with radiotherapy and limited surgery has been reported, especially in Japanese literature, but has not made significant impact in the treatment of squamous cell carcinoma [27-29]. Our study did not show any benefit of anterior chemotherapy followed by radiation in squamous cancer of maxillary antrum. It showed 5-year local control rate of only 26.3% in patients who received chemotherapy followed by radiation, as compared to 28% local control rate for radiation alone and 63.8% 5-year local control rate for a combination of surgery and radiation.

Among patients with head and neck cancer, more are alleged to die from second tumours than from their original disease [30, 31]. The risk of a second primary tumour is thought to be independent of the stage of the first tumor and seems to be no greater in men than in women, but whether it is influenced by the site of the primary tumour remains controversial [32-34]. The greatest risk, however, seems to be the continued use of alcohol and tobacco though opinion varies about which one is more influential. Wynder et al [35] found tobacco but not alcohol to be associated with an increased risk. They also report, however, that stopping both smoking and drinking did not prevent further tumours from developing, although Moore[36] showed a decreased incidence when smoking ceased. Moore also noted a greater risk of second malignant tumours with continued smoking [36]. Others also have found that smoking and drinking together seem to increase the risk of second malignant tumours [32, 34, 35]. Furthermore, the longer the patients survive, the more likely they are to develop another tumour [30, 36]. Odgen et al documented that normal oral mucosa exposed to ionizing radiation during the treatment of orofacial tumours displays abnormal DNA profiles [37]. Although these changes returned to the normal diploid state within six weeks after completing treatment, the potential for latent radiation damage remains [38-40]. This may explain why after five years the incidence of second malignant tumours seems to be greater in those who received radiotherapy [30]. The tumour itself may exercise an effect on the regional mucosa. Slaughter et al first described the concept of field cancerization [40], and research using exfoliative cytology has identified evidence for field change within the normal oral mucosa of patients with oral cancer even in those who do not smoke or drink alcohol [41]. In our study, we found second primary tumors in 19 (5%) patients. All these patients had received radiation ranging from 5500 cGy to 7000 cGy. Average interval from the time of diagnosis of primary tumor and the appearance of second primary malignancy was 7.68 years (range 4.5 to 11.5 years). Commonest location of these tumors was the gingivo-buccal area (9 patients), and floor of mouth and tonsil were least common locations (1 each site). Dorsal tongue was the second most common site with 5 tumors and larynx was the third most common site (3 tumors). No mortality was immediately associated with second primary cancers in our series. This was due to the fact that all tumors were diagnosed at a relatively early stage because of a regular follow-up.

Keeping in view the limitations of a retrospective study, a definitive recommendation can not be made based on our study. However, the results of the current study regarding the treatment of squamous cell carcinoma of the maxillary sinus show the superiority of surgery and postoperative radiation therapy as compared with preoperative radiation and surgery, radiation therapy alone or chemotherapy followed by radiotherapy. For patients who cannot undergo surgical resection, radiation therapy alone can be used; however, our results indicate a local control rate of only 28% at 5 years.
